# From Genes to Fisheries: A Synthesis of Current Research in Crustacean Biology and Management

**DOI:** 10.3390/biology14060677

**Published:** 2025-06-11

**Authors:** Yafei Duan, Lutz Auerswald, Xianliang Meng

**Affiliations:** 1 State Key Laboratory of Mariculture Biobreeding and Sustainable Goods, Yellow Sea Fisheries Research Institute, Chinese Academy of Fishery Sciences, Qingdao 266071, China; duanyafei89@163.com; 2 Laboratory for Marine Fisheries Science and Food Production Processes, Qingdao Marine Science and Technology Center, Qingdao 266237, China; 3 Key Laboratory of South China Sea Fishery Resources Exploitation & Utilization, Ministry of Agriculture and Rural Affairs, South China Sea Fisheries Research Institute, Chinese Academy of Fishery Sciences, Guangzhou 510300, China; 4 Department of Animal Sciences, Stellenbosch University, Stellenbosch 7600, South Africa; lutz.auerswald@gmail.com

Crustaceans represent an extraordinarily diverse group of arthropods, occupying a vast array of aquatic and terrestrial habitats worldwide [1]. Their ecological significance is profound, influencing food web dynamics and nutrient cycling, and serving as crucial indicators of ecosystem health [2,3]. Economically, many species are pillars of global fisheries and the rapidly expanding aquaculture sector [4]. A deep understanding of their complex biology—from their genetic architecture and physiological responses to environmental stresses and anthropogenic impacts—is essential for their effective conservation and sustainable management, particularly in the context of global environmental change.

This collection of 17 research articles presents recent advancements across the broad spectrum of crustacean biology. These contributions, from a multitude of researchers, highlight the dynamic nature of the field and the innovative approaches being employed to address both fundamental biological questions and pressing applied challenges. The studies explore critical areas, including molecular genetics and evolution, physiological adaptations to environmental stressors, innovations in aquaculture techniques, neuroendocrine control of vital life processes, fisheries science, and sensory biology.

Significant strides have been made in understanding the genetic underpinnings of crustacean life. Modern molecular tools have provided unprecedented insights into phylogenetic relationships, the genetic diversity within and between populations, and the impacts of genetic factors such as inbreeding on viability and performance [5,6,7,8,9]. These genetic studies are not only crucial for evolutionary biology but also form the bedrock for developing genetically improved strains for aquaculture, focusing on traits such as disease resistance and stress tolerance.

A substantial body of research is dedicated to elucidating how crustaceans respond and adapt to a changing environment. Investigations into the physiological and molecular responses to stressors such as fluctuating salinity, ammonia exposure, and hypercapnia have revealed the complex mechanisms involved in maintaining homeostasis [10,11,12,13,14,15,16]. Transcriptomic, proteomic, and metabolomic analyses have identified the key genes, pathways, and molecular markers associated with stress adaptation, offering a deeper understanding of crustacean resilience and vulnerability.

The aquaculture of crustaceans plays a crucial role in global food production, and research in this area is focused on enhancing sustainability and efficiency. Studies have explored the efficacy of novel dietary supplements, including seaweeds and probiotics, to improve growth, health, and disease resistance [17,18,19]. Concurrently, advancements in reproductive technologies, such as sperm cryopreservation, are critical for establishing germplasm banks, facilitating selective breeding programs, and overcoming challenges related to asynchronous maturation [20,21].

The intricate neuroendocrine systems that regulate crucial life-cycle events in crustaceans, such as molting and reproduction, continue to be an active area of investigation [22,23,24,25]. Identifying neuropeptides and their receptors and understanding their roles in these physiological processes can lead to new strategies for manipulating growth and reproduction in cultured species.

Furthermore, research into the dynamics of wild crustacean populations and their sensory biology provides essential data for informed fisheries management and conservation strategies [26]. Studies assessing stock status, life history traits, and the impacts of fishing pressure are vital for ensuring the long-term viability of commercially important fisheries. Investigations into sensory systems, such as statocysts, contribute to our understanding of how crustaceans perceive and interact with their environment.

To provide a comprehensive overview and a quick reference for readers to navigate the diverse topics covered in this Special Issue, Table 1 summarizes the 17 published studies, categorizing them by their primary research focus and highlighting their key findings and contributions.

In conclusion, this Special Issue provides a valuable and timely overview of the current state of crustacean biology. The breadth of topics and the depth of investigation highlight the vibrancy of this field. The collective findings underscore the necessity of continued research, fostering integrative and interdisciplinary approaches. Such endeavors are crucial for advancing our fundamental knowledge of these fascinating organisms and developing scientifically informed strategies to ensure the sustainable management of crustacean populations and their ecosystems in the face of ongoing and future challenges.

## Figures and Tables

**Table 1:** Summary of 17 research articles on crustacean biology, highlighting species studied, primary research focus, key findings/contribution, and authors.

Species Studied	Primary Research Focus	Key Finding/Contribution	Author(s)
*Fenneropenaeus chinensis* (shrimp)	Elimination effects of inbreeding on genotype frequency in larval stages.	Showed significant inbreeding depression in larval survival, with selection favoring heterozygotes and increasing population heterozygosity post-elimination.	Fu et al. [5]
*Fenneropenaeus merguiensis* (shrimp)	Nutritional value, antioxidant potential, and genetic diversity from three Chinese regions.	Identified a population with superior nutritional quality (ash, protein, EAAs) and relatively higher genetic diversity, highlighting its potential for aquaculture.	Li et al. [7]
*Litopenaeus vannamei* (shrimp)	Nutritional composition, physiological indicators, and genetic diversity across different aquaculture populations.	Found consistent nutritional content but significant differences in genetic diversity in different aquaculture populations.	Li et al. [6]
*Litopenaeus vannamei* (shrimp)	Genome-wide identification and expression of neuropeptides; expression patterns after CHH gene RNAi.	Identified 125 neuropeptide-encoding genes, noted gene family expansions (ACP, CHH, and PDH), and found most neuropeptide genes downregulated after CHH/VIH silencing.	Zhang et al. [23]
*Litopenaeus vannamei* (shrimp)	Development of DNA markers for Acute Hepatopancreatic Necrosis Disease (AHPND) tolerance using GWAS.	Identified four candidate SNPs and 17 InDels associated with AHPND tolerance using DArT sequencing.	Whankaew et al. [27]
*Metapenaeus monoceros* (shrimp)	Life history traits and fishery dynamics along the Saudi Arabian Red Sea coast.	Estimated growth parameters, indicated overfishing, and suggested current fishing pressure reduces spawning stock biomass to 23% of unexploited levels.	Gireesh et al. [28]
*Penaeus monodon* (shrimp)	Metabolic response to acute ammonia nitrogen stress.	Revealed severe tissue damage, conversion of ammonia to urea for detoxification, increased SOD activity, and inhibited caspase activity, indicating complex metabolic and stress responses.	Ding et al. [10]
*Penaeus monodon* (shrimp)	Regulatory role of microRNAs under moderately low salinity stress.	Identified differentially expressed miRNAs in hepatopancreas after low salinity exposure, targeting genes in metabolism, immune response, and stress adaptation pathways.	Shi et al. [13]
*Penaeus monodon* (shrimp)	Effects of dietary *Gracilaria lichenoides* and *Bacillus amyloliquefaciens* on growth, antioxidant capacity, and intestinal health.	G. lichenoides supplementation significantly improved growth performance; additives enhanced lipase activity and antioxidant gene expression and modulated intestinal microbiota.	Tian et al. [17]
*Penaeus monodon* (shrimp)	Optimization of sperm cryopreservation protocol.	Optimized protocol using 10% DMSO with natural seawater, programmable freezing, and 37 °C thawing achieved 85% sperm viability after 15 days with minor acrosomal damage.	Kong et al. [20]
*Procambarus clarkii* (crayfish)	Transcriptomic responses to different salinity stress.	Revealed metabolic pathways as primary responses, differential expression of immune/antioxidant genes, and specific signaling pathways (Foxo, Wnt, Hippo, and Notch) activated under high-salinity.	Luo et al. [11]
*Jasus lalandii* (lobster)	Influence of hypercapnia on physiology of ovigerous females and embryonic development.	Berried females efficiently respond to acute and chronic hypercapnia by increasing hemolymph bicarbonate; embryo growth and development are not impacted by chronic hypercapnia.	Ritter et al. [12]
*Nephrops norvegicus* (lobster)	Statocyst ultrastructure.	Described the fine morphology of the statocyst sensory setae using SEM and TEM, contributing to understanding sound perception.	Solé [29]
*genus Panulirus* (lobsters)	Shifts in catch composition in the artisanal spiny lobster fishery.	Revealed considerable shifts in catch composition of Spiny lobsters over time compared to 1970s data, highlighting changes in species dominance in Kenya.	Kulmiye [26]
*Portunus trituberculatus* (crab)	Role of corazonin in stimulating ecdysteroid synthesis during molting.	Demonstrated that PtCrz peptide stimulates ecdysteroid levels and related gene expression in vitro and in vivo, potentially via PtCrzR and affecting PtETH expression.	Xie et al. [22]
*Portunus trituberculatus* (crab)	Comparative transcriptome analysis of response to *Vibrio parahaemolyticus* and low-salinity stress.	Identified differentially expressed genes involved in ion transport, immunoregulation, apoptosis, and Hippo signaling pathway under dual stress conditions.	Sun et al. [14]
*genus Tuerkayana* (crabs)	Molecular phylogeny and evolution based on whole mitochondrial genome sequences.	Provided mitochondrial evidence for *Tuerkayana*, clarified Gecarcinidae genera, and identified selective pressure in the *nad6* gene.	Wang et al. [30]
